# Role of *Rhipicephalus bursa* larvae in transstadial transmission and endemicity of *Babesia ovis* in chronically infected sheep

**DOI:** 10.3389/fcimb.2024.1428719

**Published:** 2024-07-26

**Authors:** Recep Firat, Mehmet Can Ulucesme, Munir Aktas, Onur Ceylan, Ferda Sevinc, Reginaldo G. Bastos, Carlos E. Suarez, Sezayi Ozubek

**Affiliations:** ^1^ Department of Parasitology, Faculty of Veterinary Medicine, University of Firat, Elazig, Türkiye; ^2^ Department of Parasitology, Faculty of Veterinary Medicine, University of Selcuk, Konya, Türkiye; ^3^ Animal Disease Research Unit, Agricultural Research Service, US Department of Agriculture, Pullman, WA, United States; ^4^ Department of Veterinary Microbiology and Pathology, College of Veterinary Medicine, Washington State University, Pullman, WA, United States

**Keywords:** *Babesia ovis*, ELISA, experimental infection, PCR, *Rhipicephalus bursa*, sheep

## Abstract

*Babesia ovis*, transmitted by *Rhipicephalus bursa* ticks, is the causative agent of ovine babesiosis, a disease characterized by fever, anemia, hemoglobinuria, and high mortality in sheep. This study investigates whether sheep that survived babesiosis without treatment can serve as a source of infection for *B. ovis*-free host-seeking *R. bursa* larvae in a later season. Three donor sheep were experimentally infected with *B. ovis*, and after six months, persistence of *B. ovis* was assessed through blood and tick transmission experiments. Blood from donor sheep was intravenously injected into three recipient sheep, while donor sheep were also infested with *B. ovis*-free *R. bursa* larvae. Engorged nymphs molted to adults, and new recipient sheep were infested with these ticks. All recipient sheep were monitored for *B. ovis* for 100 days using microscopic, serological, and molecular approaches. The presence of *B. ovis* was confirmed in the recipient sheep that received blood, leading to clinical infection in two. However, no *B. ovis* was detected in recipient sheep infested with ticks. These results suggest that sheep recovering from *B. ovis* infection do not serve as a source of infection for *R. bursa* larvae in subsequent seasons.

## Introduction

Babesiosis is a zoonotic protozoal disease caused by *Babesia* species, commonly observed in domestic and wild animals (Iqbal et al., 2011; Schnittger et al., 2022). Species within the *Babesia* genus, such as *B. ovis*, *B. motasi*, *B. crassa*, *B. foliata*, and *B. taylori* have been reported to cause babesiosis in sheep and goats (Schnittger et al., 2022). Additionally, new species or genotypes, including *Babesia* sp. Xinjiang and *Babesia* sp. BQ1 (Lintan) in sheep, as well as *Babesia aktasi* n. sp. in goats, have been reported (Guan et al., 2009, 2010; Ozubek and Aktas, 2017; Ozubek et al., 2023a). *Babesia ovis*, the primary causative agent of babesiosis in small ruminants, causes significant economic losses in sheep in tropical and subtropical regions. As an endemic ovine pathogen in South Europe, Africa, the Middle East, and Asia, *B. ovis* infection manifests clinically with clinical signs characterized by fever, hemolytic anemia, and hemoglobinuria, often leading to fatalities (Yeruham et al., 1998; Ceylan et al., 2021). The epidemiology of *B. ovis* is closely linked to the biology and ecology of *Rhipicephalus* ticks, particularly *R. bursa* (Yeruham et al., 1998). Both immature (larvae, nymphs) and adult stages of *R. bursa* primarily prefer sheep and goats as their hosts (Yeruham et al., 1995; Altay et al., 2008). *Rhipicephalus bursa*, a two-host tick that completes only one generation per year, derives its name from the Latin term “bursa” referring to the bloated pouch-like appearance of engorged females (Walker et al., 2000). This tick species exhibits a life cycle ranging from 99 to 214 days under laboratory conditions. The extended lifespan may be associated with the survival capacity of unfed larvae in warm and dry climate conditions. Adult *R. bursa* ticks become active when the average daytime and nighttime temperatures exceed 18°C and 12°C, respectively (Yeruham et al., 2000). *Rhipicephalus bursa* larvae remain inactive during the 5-6 months of warm and dry summer. Infestation on their hosts begins in October, peaks in December, and shows a slow decline until February. Unfed larvae and engorged nymphs of *R. bursa*, respectively, enter their states of inactivity or diapause during both the summer and winter. Sheep babesiosis cases are known to peak between May and July when adult *R. bursa* ticks are most abundant, followed by a decline and complete disappearance by the end of July (Yeruham et al., 1989, 1995). *Babesia ovis*, belonging to the *Babesia* sensu stricto group, is transmitted by vector ticks both transtadially and transovarially (Jalovecka et al., 2019). In competent vector ticks, acting as definitive hosts, *B. ovis* parasites undergo sexual reproduction, ultimately leading to transovarial transmission. Thus, different life stages of the parasite, including gametocytes, zygotes, kinetes, sporoblasts, and sporozoites can be present in infected ticks. *Rhipicephalus bursa* acquires the pathogen during the larval and nymphal stages (Erster et al., 2016) and transmits the pathogen to susceptible hosts during the adult stage (Yeruham et al., 1998; Erster et al., 2016). Transovarial transmission occurs upon invasion of ovary and eggs by the parasite in engorged female ticks (Buscher et al., 1988). A study on the transmission of *B. ovis* by *R. bursa* (Erster et al., 2016) reported that infected *R. bursa* larvae and nymphs, despite feeding on susceptible hosts, do not transmit *B. ovis*, and the parasite was only transmitted to the susceptible host by adult ticks. Therefore, clinical infections resulting from *B. ovis* are expected to be prevalent during the peak activity of adult *R. bursa* ticks, which occurs between May and July (Yeruham et al., 1998).

Despite numerous studies on the prevalence of *B. ovis*, several aspects of the transmission dynamics of the disease and factors influencing its epidemiology remain unknown. The objective of this study is to investigate whether sheep that have naturally overcome *B. ovis* infection without treatment during the peak clinical period (May to July) can serve as a source of infection for *R. bursa* larvae during the active host-seeking period between October and December (Fall season). This research aims to elucidate aspects of the transmission dynamics of *B. ovis* and contribute to the understanding of factors influencing its epidemiology, particularly the potential for sheep to act as reservoirs for the pathogen outside the peak infection season.

## Materials and methods

### Ethics statement

This study was carried out according to the regulations of animal and welfare issued by the Turkish legislation for the protection of animals. All animal experiments were approved by the Firat University, Animal Experiment Ethic Committee, protocol number 2023/12-05.

### Sheep

*Babesia*-, *Anaplasma*-, and *Theileria*-free sheep were used in this study. To select experimental animals, blood samples (serum and EDTA tubes) were collected from apparently healthy 5-8-month-old sheep (n=10). Nested PCR with general primers, including Ec9/Ec12A (Kawahara et al., 2006)-16S8FE/B-GA1B (Bekker et al., 2002) for *Anaplasma*, and Nbab1F/Nbab1R (Oosthuizen et al., 2008)-RLBF2/RLBR2 (Georges et al., 2001) for *Babesia* and *Theileria*, were used to investigate the presence of these species. Samples that tested negative by nested PCR were subsequently subjected to indirect ELISA (iELISA) using BoSA1 protein for *B. ovis* specific antibody detection (Sevinc et al., 2015). Ten sheep determined to be negative by both nPCR and iELISA were included in the study. During the experiment, the sheep were relocated to a separate compartment at the Elazig Veterinary Control Institute Directorate. They were kept in a tick-free environment and provided with feed and water *ad libitum*.

### Experimental infection

*B. ovis*/Alacakaya stabilates stored in a cryobank were utilized for experimental infection. The stabilates were thawed and promptly administered intravenously to one splenectomized sheep. The splenectomy was carried out using standard surgical, anesthetic, and analgesic procedures, as described by Sevinc et al. (2007). Once the parasitemia level reached 5%, 30 ml of infected fresh blood obtained from the donor splenectomized sheep were intravenously inoculated into each of the three donor spleen-intact sheep (#021, #668, and #671), as previously described (Guan et al., 2009; Ozubek et al., 2023b). Blood was collected from experimental donor animals for microscopy, nPCR and iELISA analyses daily during the first 40 days after experimental infection and then weekly up to 6 months after infection.

### Microscopic detection of piroplasm

Blood smears were subjected to staining using a 10% Giemsa solution. The stained smears were then observed under a 100X objective lens to identify intraerythrocytic parasites. The percentage of parasitized erythrocytes (PPE) was determined by analyzing a minimum of 20 microscopic fields, as outlined in the methodology by Sevinc et al. (2007).

### *Babesia ovis* nested PCR

Genomic DNA was extracted from 200 µL of EDTA anticoagulated blood samples obtained from sheep, as well as from *R. bursa* ticks used in experimental infestations, utilizing the PureLinkTM Genomic DNA Mini Kit (Invitrogen Corporation, Carlsbad, USA) following the manufacturer’s guidelines. Subsequently, the isolated DNA was stored at −20°C until further use. To identify *B. ovis* DNA, a nPCR assay was conducted utilizing the following sets of primers, Nbab1F/Nbab1R (Oosthuizen et al., 2008) and BboF/BboR (Aktas et al., 2005). To check the DNA of *R. bursa*, PCR was conducted using 16S + 1 and 16S – 1 primers (Black and Piesman, 1994), as previously described.

### *Babesia ovis* indirect ELISA

Expression and purification of recombinant *B. ovis* Secreted Antigen-1 (rBoSA1) were carried out following the procedure outlined by Sevinc et al. (2015). Briefly, 50 µl of the reconstituted rBoSA1, prepared at a concentration of 2 μg/ml in carbonate-bicarbonate buffer (pH=9.6), was added to individual wells in a 96-well ELISA microplate. The subsequent test procedures were conducted in accordance with the methodologies previously detailed by Sevinc et al. (2015). The cutoff value was determined by adding the average value to three times the standard deviation of the optical density (OD) measurements obtained from the negative sheep sera.

### Investigation of *in vivo* persistence of *B. ovis* following experimental infection

Following experimental infection, sheep were monitored for clinical signs of ovine babesiosis, including increased body temperature, anemia, jaundice, and hemoglobinuria. Additionally, the presence of *B. ovis* was tracked in the infected sheep by nPCR and iELISA by a period of 200 days (Aktas et al., 2005; Sevinc et al., 2015).

### Transmission by direct blood inoculation

**Figure 1:**
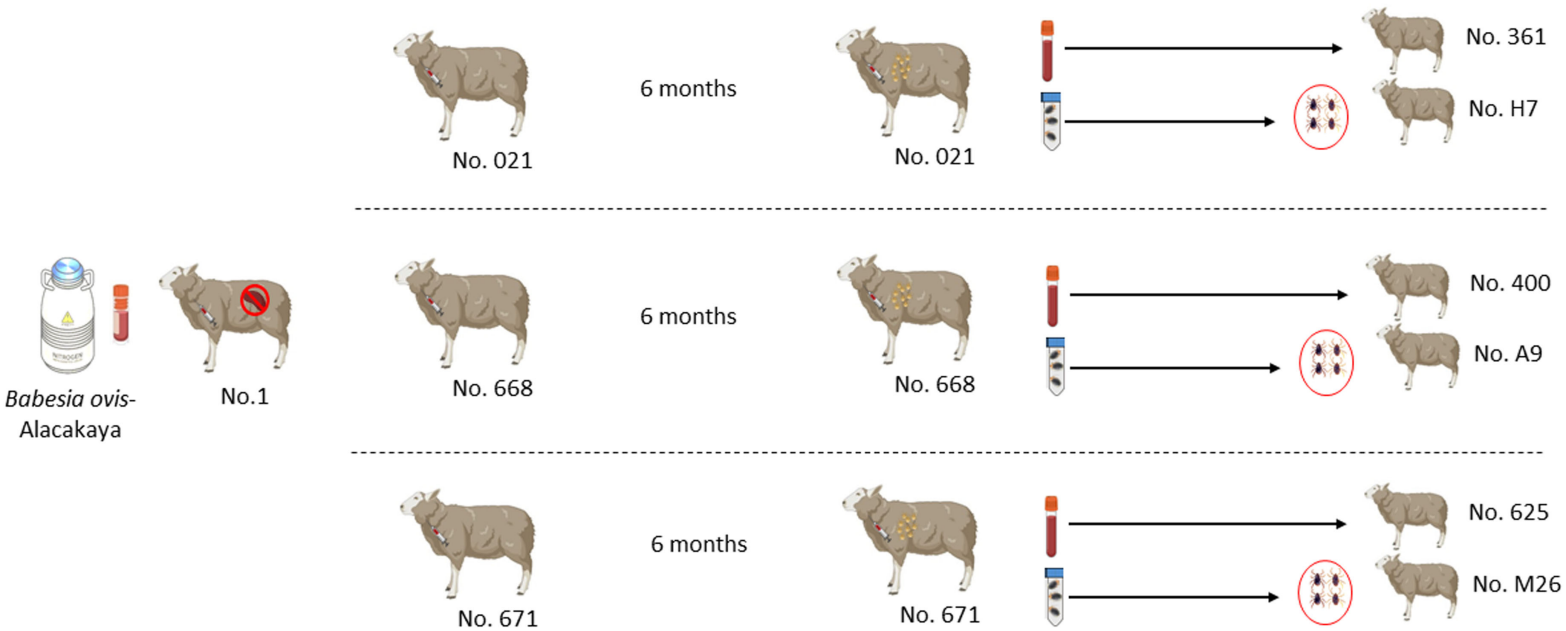
Schematic representation of the experimental design sheep used in experimental infection with blood and ticks.

Six months after the experimental infection, 100 mL of blood was collected from each donor sheep into EDTA tubes, and the individual blood was transfused into individual recipient sheep free from tick-borne pathogens (Schwint et al., 2009). Specific identification of donor (#021, #668, and #671) and recipient (#361, #400, and #625) sheep are described in **Figure 1** and **Table 1**. Sheep infected through blood transfusion (#361, #400, and #625) were monitored for babesiosis through microscopy, iELISA, and nPCR methods for 100 days post-infection (Schwint et al., 2009).

**Table 1:** Donor sheep, transmission approach via blood or ticks, and recipient sheep.

Transmission by direct blood inoculation		
Donor sheep	Inoculation amount	Recipient sheep
No: 021	100 ml-intravenous	No: 361
No: 668	100 ml-intravenous	No: 400
No: 671	100 ml-intravenous	No: 625
Transmission by tick feeding		
Donor sheep	*R. bursa* adult tick	Recipient sheep
No: 021	50 female 50 male	No: H7
No: 668	50 female 50 male	No: A9
No: 671	50 female 50 male	No: M26

### Transmission by tick feeding

*pRhipicephalus bursa* ticks employed for experimental infestations were obtained from a colony regularly cultivated within our laboratory. Briefly, to establish the colony, we collected engorged female *R. bursa* ticks from cattle and placed them in an incubator to lay eggs. After the oviposition process was completed, the egg cluster was divided into two parts, with one half used for DNA extraction. The eggs were examined for the presence of *Babesia*/*Anaplasma* using nPCR (Ozubek et al. 2023b), and the clusters found to be negative were used to obtain larvae. These larvae were then used to infest a splenectomized sheep. Engorged nymphs collected from the splenectomized sheep were placed in an incubator to obtain unfed adults. These unfed adults were then used to infest another splenectomized sheep, and the engorged females were incubated to produce larvae. This process was repeated for three generations, and each time the larval clusters were examined for the presence of *Babesia*/*Anaplasma* using nPCR. After these procedures, the colony was considered *Babesia*-free. The ticks utilized in the transmission experiments were derived from eight successive generations bred and raised under laboratory conditions in our facility. Additionally, this colony has been extensively employed in our laboratory to transmit *B. ovis* (Sevinc et al., 2023). Six months after experimental infection, *B. ovis*-free unfed larvae (0.1 g for each sheep) were administered to the donor sheep (#021, #668, and #671) within a capsule. Engorged nymphs were collected from each sheep in separate tubes, placed in an incubator to molt into adults. The obtained unfed adult ticks (50 females and 50 males for each sheep) were transferred to recipient sheep free from tick-borne pathogens (#H7, #A9, and #M26). (**Figure 1** and **Table 1**). For transstadial transmission, sheep (#H7, #A9, and #M26) were monitored for babesiosis through microscopy, iELISA, and nPCR methods for 100 days post-infestation (Schwint et al., 2009).

## Results

### Long-term monitoring and infection dynamics of *B. ovis* infection in sheep

**Figure 2:**
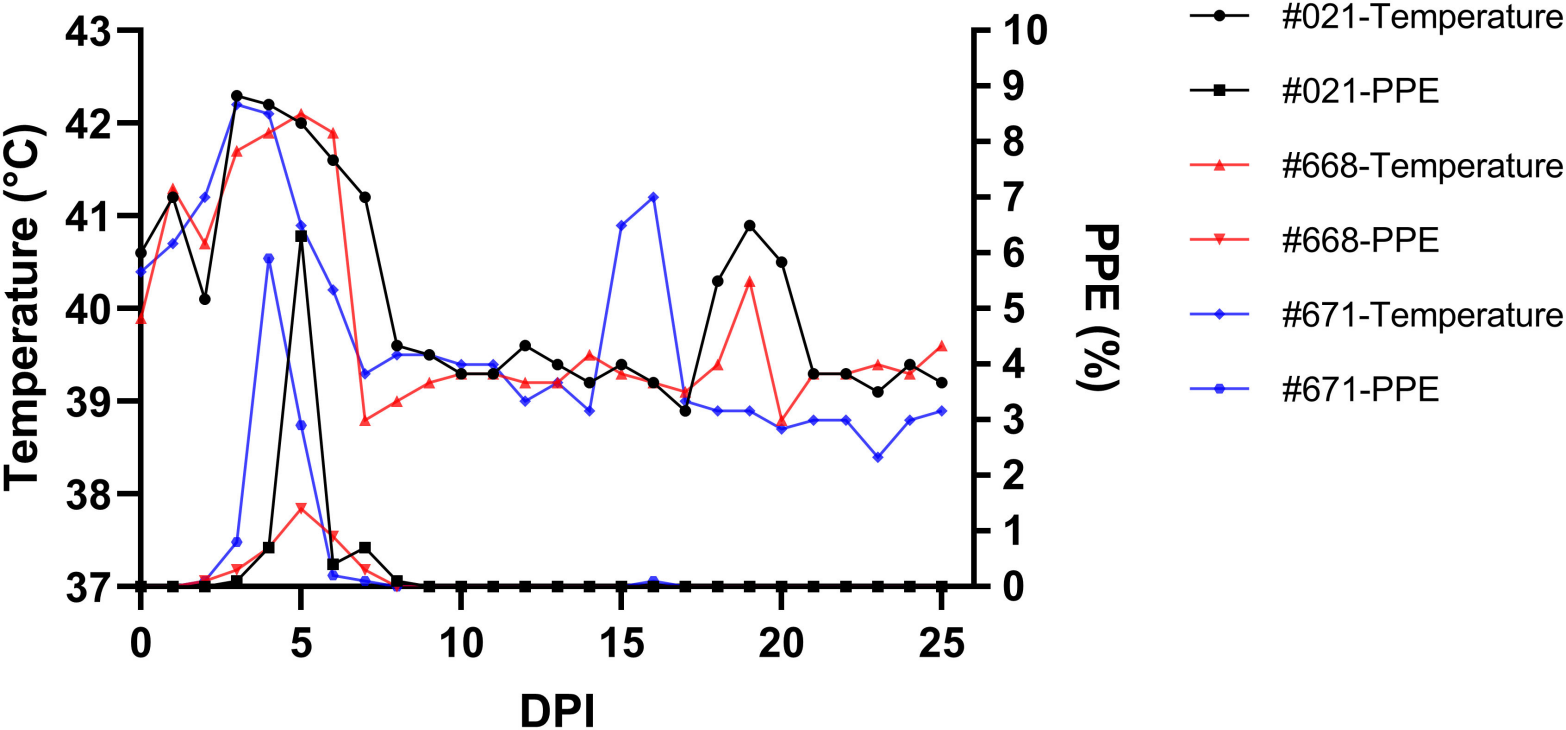
Monitoring parasitemia (PPE) and body temperature in sheep (#021, #668, #671) during the day post-infection period.

Parasites were identified in peripheral blood between the second and third days following the experimental infection of sheep with *B. ovis*/Alacakaya stabilate. As the infection progressed, all sheep exhibited fever, reaching peak temperatures between 42.0°C and 42.3°C, which corresponded to an increase in parasitemia. Microscopic examination of peripheral blood smears indicated parasitemia rates ranging from 5.9% to 6.3%. Severe clinical signs of babesiosis, such as hemoglobinuria, jaundice, decreased appetite, and lethargy, were observed in all experimentally infected sheep. Additionally, these sheep exhibited a significant decrease of >20% in the hematocrit levels. Parasitemia persisted for 6-7 days in all three sheep. Remarkably, despite severe babesiosis clinical signs, all sheep successfully recovered from the disease without treatment. In one sheep (#671), 9 days after the end of parasitemia, the temperature rose to 41.2°C, and piroplasms were observed in the blood smear. The following day, the fever in this sheep decreased to 39.0°C, and no piroplasms were detected under the microscope (**Figure 2**; **Table 2**).

**Table 2:** Changes in infection parameters in sheep day post infection (DPI).

Animal ID	Prepatent period	Max. parasitemia	Max. fever	Duration of fever days	Relapse (DPI)
021	3 DPI	6.3%	42.3°C	7	–
668	2 DPI	1.4%	42.1°C	6	–
671	2 DPI	5.9%	42.2°C	6	+ (16)

**Figure 3:**
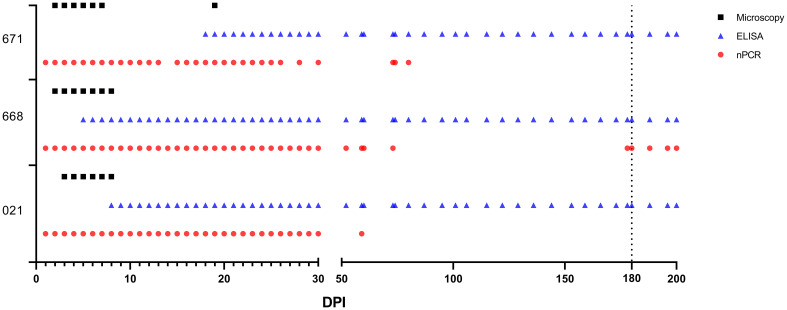
*Babesia ovis* infection dynamics by microscopy, nPCR and iELISA. On day 180, experimental animals were infested with sterile *R. bursa* larvae, and the blood taken from these animals was administered to the recipient animals.

One day after experimental infection, the presence of the parasite in circulation was demonstrated through nPCR performed on DNA isolated from experimentally infected sheep’s blood. In sheep #021, #668, and #671, the presence of the parasite varied, continuing until days 59, 200, and 80, respectively, following experimental infection. Furthermore, in each of the three sheep, the parasite showed a fluctuating pattern of testing either as positive or negative after 50 days post-infection (DPI) (**Figure 3**).

**Figure 4:**
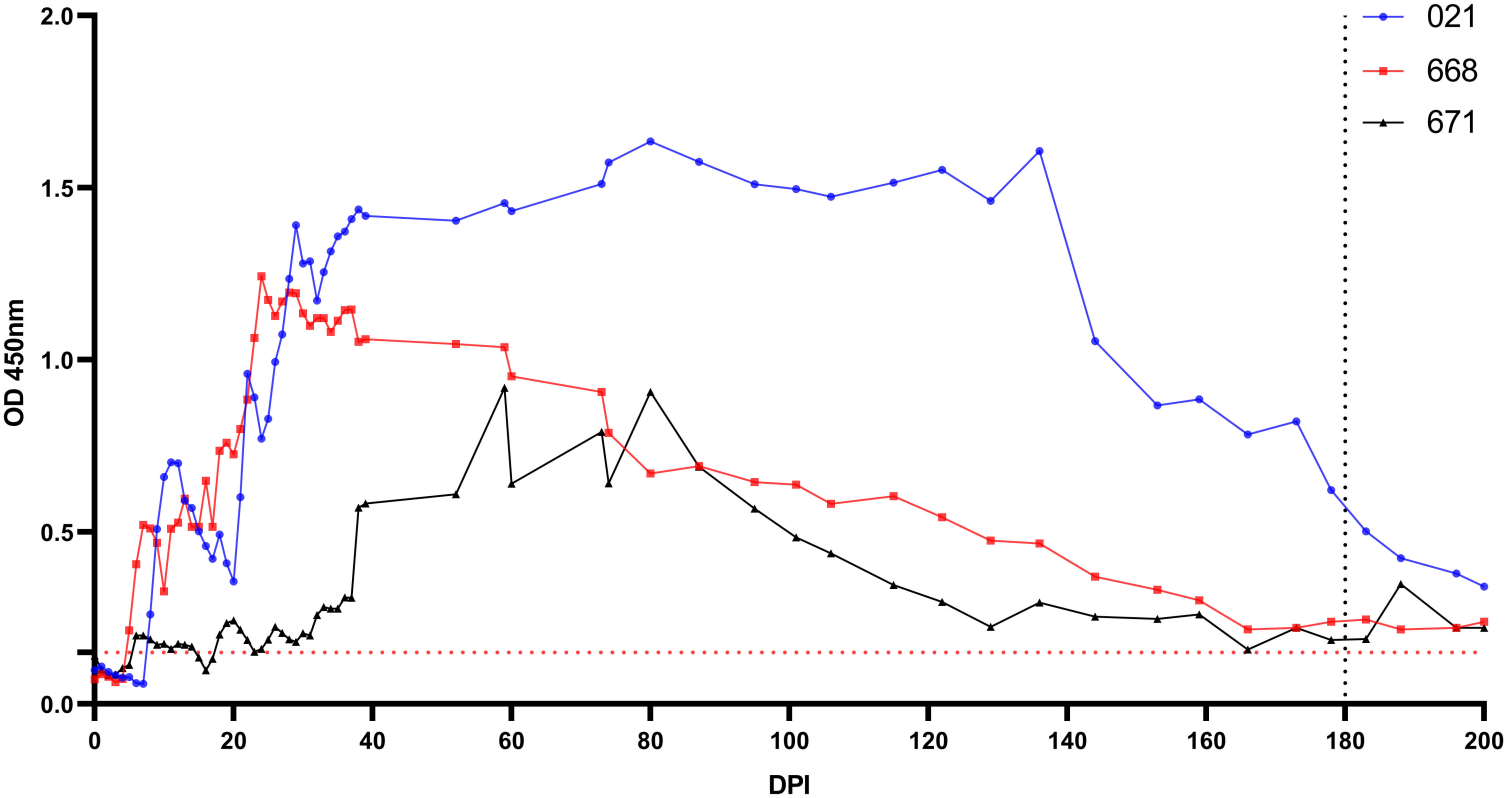
Antibody detection by iELISA for the detection of *B ovis* using rBoSA1 antigen on experimental animals (#021, #668, and #671). The Y and X axes represent absorbance at 450 nm and days post experimental infection (DPI) (days 0–200), respectively. Day 180 shows experimental infections. The red dashed line represents the cutoff value of the iELISA, calculated as the OD of non-infected sheep sera plus 3 standard deviations.

Antibodies were detectable in experimentally infected sheep starting from 5-18 DPI. Following the initial detection, specific antibody responses to *B. ovis* persisted in all three sheep for more than 6 months after experimental infection (**Figure 4**). Fluctuations observed in nPCR were absent in the rBoSA1 iELISA.

**Figure 5:**
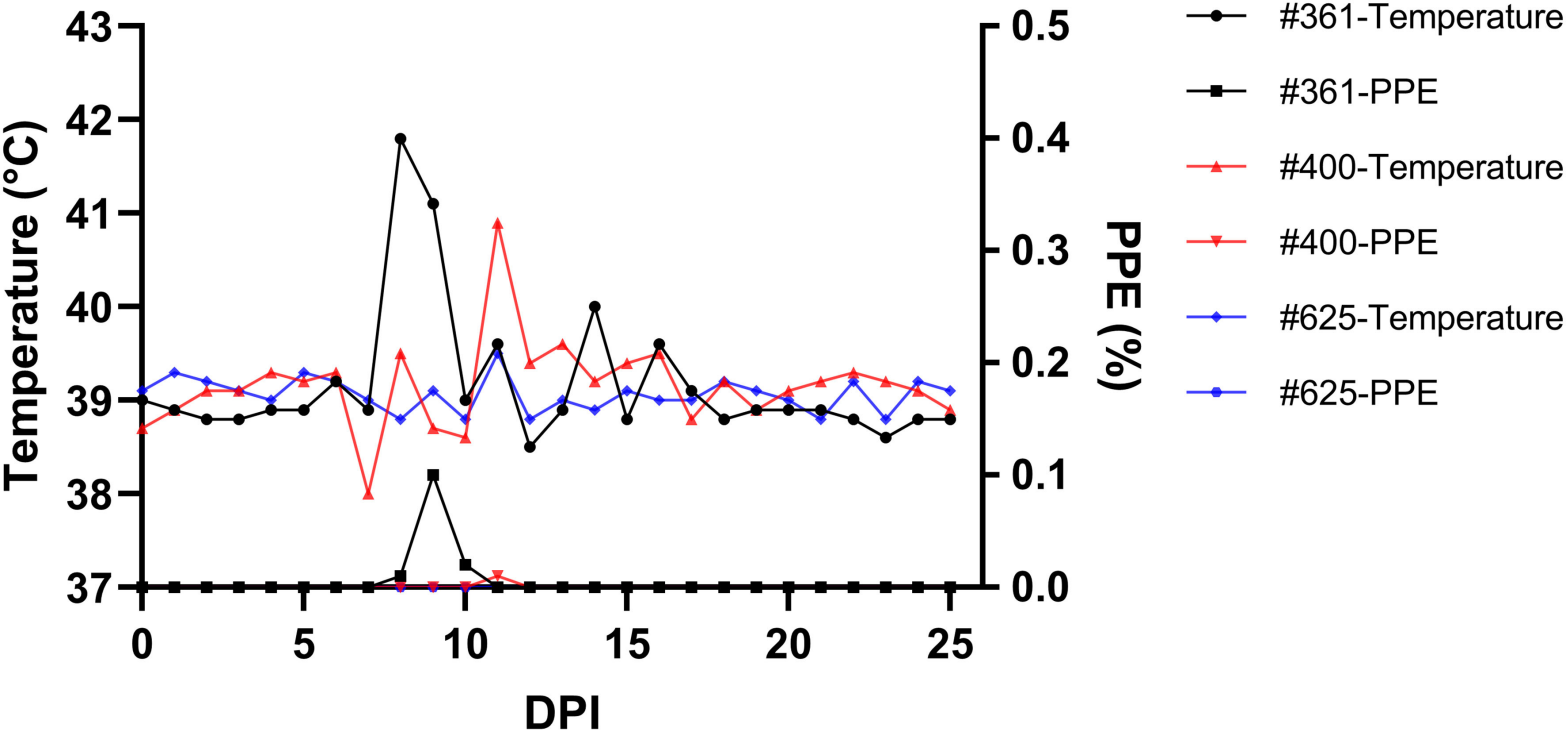
Monitoring parasitemia (PPE) and body temperature in sheep (#361, #400, #625) during the day post-infection period.

**Figure 6:**
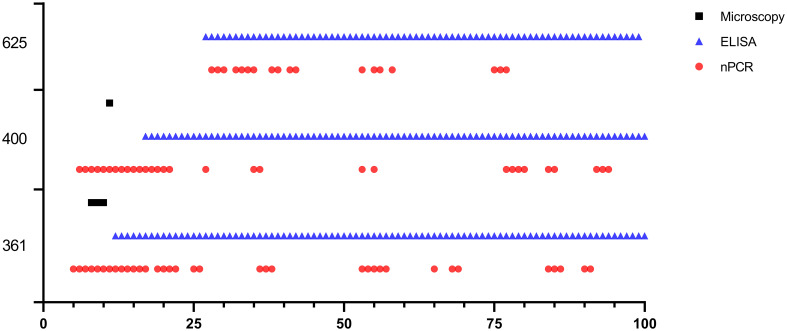
Long-term monitoring of sheep #361, #400, and #625 after experimental infection by microscopic examination, nPCR and iELISA.

**Figure 7:**
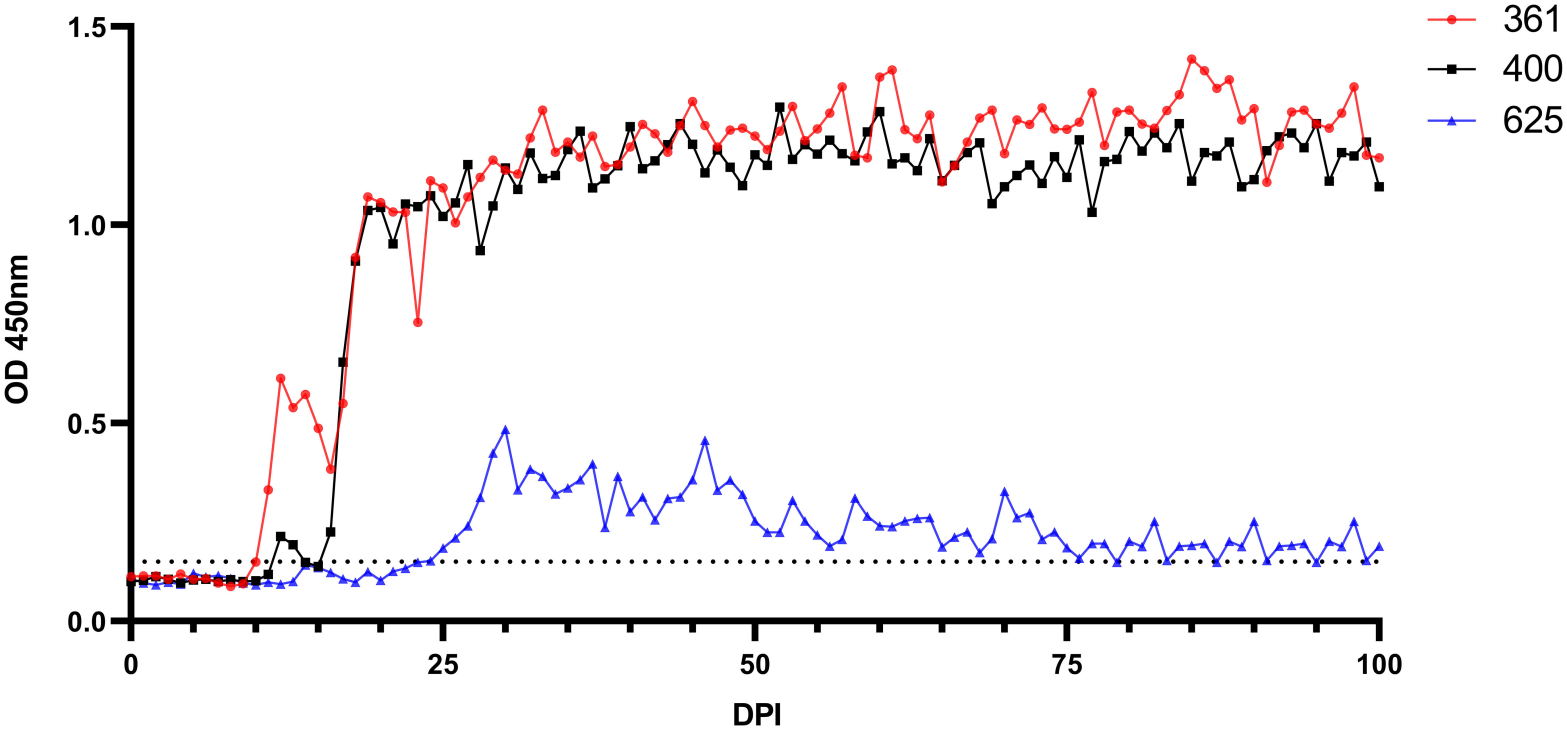
Serological analysis by iELISA for the detection of anti-*B. ovis* antibodies using rBoSA1 antigen on experimental animals (#361, #400, and #625). The Y and X axes represent absorbance at 450 nm and days after experimental infection (days 0–100), respectively. The black dashed line represents the cutoff value of the iELISA, calculated as the OD of non-infected sheep sera plus 3 standard deviations.

*Babesia ovis* transmission via blood inoculation from chronically infected sheep was performed as depicted in **Figure 2**. At the end of 6 months, nPCR results indicated the presence of *B. ovis* DNA only in sheep #668. In addition, blood transfusions were conducted to demonstrate the presence of *B. ovis* in the inoculated sheep. For this purpose, 100 ml of blood samples from #021, #668, and #671 were sequentially administered to sheep #361, #400, and #625, respectively, six months after experimental infection. Subsequently, the sheep receiving blood transfusion were monitored for 100 days using microscopic examination, nPCR, and iELISA. Following experimental infection, the initial presence of the *B. ovis* was observed in blood smears from #361 on the 7^th^ day, and parasitemia persisted until day 10 post-infection. The maximum temperature measured on #361 was 42.1°C, and the high fever endured for 3 days, with no clinical signs other than fever noted. *Babesia ovis* was detected by nPCR between days 5 and 91 post-infection, showing fluctuations during this period. Antibodies against *B. ovis* were first detected on day 12 in sample #361 and continued to be present until day 100. In #400, *B. ovis* was observed microscopically at 8 DPI, and parasitemia lasted only for one day. A maximum temperature was recorded as 41.7°C, and like #361, no clinical signs other than high fever were observed. Following infection, the presence of *B. ovis* was detected by nPCR between days 6 and 94, with fluctuations during this period. In #625, the first positive reaction with iELISA started on day 17 and continued until day 100 post-infection. Unlike the other two sheep, in #625, no microscopic or clinical signs were observed after blood transfusion. Following infection, the presence of *B. ovis* was demonstrated by nPCR between days 28 and 77, with fluctuations during this period. The first positive reaction with iELISA started on day 27 and continued until day 100 (**Figures 5**–**7**; **Table 3**).

**Table 3:** Monitoring results of *B. ovis* infection in sheep following blood transfusion.

Sheep ID	Blood Donor ID	Microscopy (DPI)	Max. Temp.	Clinical signs	nPCR Detection Period (Days)	iELISA Detection Period (Days)
#361	#021	7-10	42.1°C	High fever	5-91	12-100
#400	#668	8	41.7°C	High fever	6-94	17-100
#625	#671	None	39.5°C	None	28-77	27-100

### Sheep that survived *B. ovis* infection are not source of parasites for *R. bursa* transstadial transmission

Six months after experimental infection, sheep #021, #668, and #671 were infested with 0.1 grams of sterile *R. bursa* larvae, and after that, tick development was monitored daily. Twelve to eighteen days after experimental infestation, all engorged nymphs were collected. A total of 375, 190, and 150 engorged nymphs were collected from sheep #021, #668, and #671, respectively. From these nymphs, 302, 141, and 118 unfed adult *R. bursa* were obtained, respectively. Unfed adult ticks from individual sheep were segregated into separate pools of females and males, resulting in 12 pools (6 female, 6 male pools), each containing 5 females and 5 males (30 female, 30 male). These pools underwent nPCR analysis to detect the presence of *B. ovis*, with all pools yielding negative results. Following that, adult ticks, consisting of 50 females and 50 males per sheep, were applied to sheep #H7, #A9, and #M26 to investigate transmission. All ticks were subsequently collected from these sheep between 8 and 16 days after infestation. The transmission sheep (#H7, #A9, and #M26) were monitored for 100 days the presence of *B. ovis* using microscopic examination, nPCR, and iELISA, and the results were negative. Additionally, engorged male and female ticks collected from sheep #H7, #A9, and #M26 were individually examined with nPCR, revealing that these ticks were also negative. Collectively, these findings suggest no evidence of tick acquisition, nor transmission, of *B. ovis* from chronically infected sheep (6 months of infection) (#021, #668, and #671) to naive sheep (#H7, #A9, and #M26).

## Discussion

Ovine babesiosis, caused by *B. ovis*, is a highly significant tick-borne disease prevalent in the Mediterranean region and other endemic areas worldwide where the vector tick *R. bursa* is present. This tick species, a two-host tick, is the exclusive known vector responsible for transmitting *B. ovis* among sheep populations (Yeruham et al., 1995, 2001; Erster et al., 2016). Extensive research has been conducted on the prevalence of *B. ovis*, yet significant gaps remain regarding the dynamics of the disease transmission and factors influencing its epidemiology. Morbidity and mortality caused by *B. ovis* typically occur once a year during the peak of adult *R. bursa* tick activity in the Spring months (April-June). Peak incidence is in May, lasting through April to July when adult ticks are active. No cases of babesiosis occur during the Autumn (September-November) when the immature stages of *R. bursa* (larvae and nymphs) are active (Yeruham et al., 1995). In this study, we utilized a sheep experimental model to investigate whether sheep infected with *B. ovis* during the peak transmission season could serve as reservoirs, potentially transmitting infection to host-seeking larval and nymphal stages of *R. bursa*.

The experimental infection of sheep with *B. ovis*/Alacakaya stabilate resulted in parasitemia in peripheral blood between 2 and 3 DPI, which was accompanied by fever. Severe clinical signs of babesiosis, including hemoglobinuria, icterus, reduced appetite, and lethargy, were also observed, leading to a substantial decline of 20% in the hematocrit level. Remarkably, the three experimentally infected sheep recovered from the acute disease without treatment, with parasitemia persisting for 6-7 days in all cases. These findings are consistent with previous results in sheep experimentally infected using the *B. ovis*/Extremadura isolate (Habela et al., 1990). However, in another study, *B. ovis*/Israel strain, parasitemia remained for 16 days in experimentally infected sheep (Erster et al., 2016). Microscopic analysis of peripheral blood smears revealed parasitemia levels ranging from 5.9% to 6.3%, indicating a high parasitemia rate based on the classification by Sevinc et al., 2013. Additionally, one sheep showed a transient rise in temperature and the presence of piroplasms in blood smear 9 days after parasitemia had finished. This result was expected, considering that parasite relapses in babesiosis have been documented in humans (Renard and Ben Mamoun, 2021), cattle (Mahoney and Mirre, 1977; Mahoney et al., 1979), and dogs (Ayoob et al., 2010). Furthermore, relapses have been reported in *B. ovis* despite imidocarb dipropionate treatment (Sevinc et al., 2007). Studies indicate that *B. bovis* infected cattle may also experience episodes of subclinical recrudescence that can happen up to 4 years after infection, likely attributed to antigenic variation (Mahoney et al., 1973). Additionally, stress associated with animal management has been identified as a potential trigger for these relapses (Stuen, 2020).

Notably, nPCR demonstrated the presence of the parasite in peripheral blood of the three sheep inoculated with blood from the donor animal, with parasitemia varying to up to 200 DPI, characterized by intermittent negative and positive results. Similar study, following experimental infection of bovines with *B. bovis*, the parasite presence was tracked using nPCR, revealing fluctuations akin to those observed in our investigation (Suarez et al., 2012; Chung et al., 2017). It was underscored that diagnostic methods reliant on molecular techniques, such as PCR, demonstrate fluctuations in parasitemia which can be attributed to parasite sequestration and antigenic variation (Suarez et al., 2012; Chung et al., 2017). While sequestration is not recognized as a characteristic of *B. ovis*, a gap on research addressing this aspect exists. Furthermore, in experimental infections conducted through blood inoculation and infected ticks, *B. ovis* was detected by qPCR for up to 180 and 395 DPI, respectively (Erster et al., 2016). Specific antibody responses to BoSA1 were detectable in all infected sheep starting at 5 to 18 DPI, consistently persisting for over 6 months post-infection. These results are in line with high antibody titers reported between days 7 and 75 after experimental infection in the rBoSA1 iELISA (Sevinc et al., 2015). The specific antibody titer, determined through IFAT, remained positive for up to 180-200 days post exposure in sheep infected with blood or tick infestation (Erster et al., 2016). Similarly, it is known that the antibody response persists for 330 days after experimental infection with *B. ovis* (Habela et al., 1990).

In the present study, *B. ovis* was exclusively detected in one sheep by nPCR six months after infection. Subsequent blood transfusions from donor sheep to recipient sheep were performed to demonstrate *B. ovis* infection. Notably, one recipient sheep exhibited parasitemia and high fever from day 7 to day 10, with *B. ovis* detected by nPCR and iELISA. Another recipient sheep exhibited a shorter parasitemia duration (one day) and a fever response similar to the first recipient. Conversely, a third recipient sheep showed no clinical signs, although *B. ovis* presence was confirmed by nPCR and antibodies to BoSA1 between days 28-77 and 27-100 post-infection, respectively. These results highlight the diverse responses among the transfused sheep following *B. ovis* infection, underscoring the intricate dynamics of this infection and suggesting that individual variability may significantly influence our findings. Several potential explanations for the distinct responses in recipient animals can be considered. First, variations in the number of parasites inoculated during stabilate or primary infection (dose) could play a crucial role in pathogenesis. Second, the animal remained persistently infected after six months may exhibit antigenic and phenotypic differences compared to the initial stabilate population. This variation could arise due to selective pressures exerted by the host’s immune system during the infection, potentially leading to the emergence of attenuated strains. These strains might already exist as a subpopulation within the original strain, subsequently selected during infection and immunity, or could arise through other processes, including epigenetic mechanisms. Additionally, the presence of genes, such as *Babesia* “vesa-like” in the genome of the parasite, could enable parasites to persist through antigenic variation mechanisms, although the specifics of how this mechanism operates in *B. ovis* remain unclear. Ultimately, whether such selection results in attenuated or phenotypically distinct parasites also remains an open question. While ticks are the main vectors for babesiosis, the disease can also spread to humans through blood transfusion. For instance, *Babesia microti* is reported as the leading pathogen transmitted via blood transfusion in the United States. Donors should be PCR screened for babesiosis before blood collection, but a negative result doesn’t rule out infection as parasite levels may be below detectable levels (Levin and Krause, 2016). Findings from our study provide supporting evidence for this phenomenon. Our research substantiates the notion that babesiosis can be transmitted despite negative nPCR results. Although blood transfusion has made significant advancements in recent years in veterinary medicine (Kumar, 2017), it is not widespread in practical terms for sheep.

It has been experimentally shown that immature *R. bursa* can acquire the agent from sheep acutely infected with *B. ovis* and transmit *B. ovis* transstadially to other sheep at the adult stage (Erster et al., 2016). However, in our study, considering natural conditions, sheep (#021, #668, #671) chronically infected with *B. ovis* six months post-acute infection were infested with immature *R. bursa*, and it was observed *in vivo* that the pathogen was not transmitted to the next generation. Several factors could explain the observed failure of transstadial transmission in our study. Firstly, the donor animals (#021, #668, #671) exhibited parasite loads that were undetectable even with nPCR, which may have contributed to the unsuccessful transstadial transmission. Experimental studies have demonstrated a direct correlation between the level of parasitemia in sheep and the number of kinetes in the tick’s hemolymph (Yeruham et al., 2001). This suggests that insufficient parasitemia in the donor animals could directly impact the ability of ticks to acquire and transmit the pathogen. Furthermore, the low parasite load in the host may influence the survival and replication of the pathogen within the tick due to interactions with the tick’s immune system. The innate immune responses of the tick, including antimicrobial peptides and phagocytic cells, could actively inhibit the transmission of *B. ovis* (de la Fuente et al., 2017). Overall, the combination of low parasitemia in donor animals, and the complex interactions between the pathogen, tick immune responses, and host immune responses appears to significantly hinder the transstadial transmission of *B. ovis* under natural conditions.

In conclusion, our study provides valuable insights into the epidemiology of *B. ovis* in sheep, particularly regarding the potential role of recovered chronically infected sheep as sources of infection for *R. bursa* larvae. While our blood transmission experiment demonstrated the presence of the parasite in recipient sheep, the tick transmission experiment indicated a lack of successful transmission from experimentally infected hosts to sterile *R. bursa* larvae and nymphs. Although generated in an experimental model, these findings suggest that sheep surviving babesiosis without treatment between May and July may not significantly contribute to the transmission of *B. ovis* to host-seeking *R. bursa* larvae in November-December. However, it should be remarked that these observations may not apply to the case of sheep acutely infected with *B. ovis*, where tick acquisition and transstadial mechanisms are more likely to occur due to a higher number of parasites in the blood intake by the ticks, which adds to the lack of selection of “attenuated” strains by the action of the immune system of the hosts at this stage of infection. Altogether, the data reported hereby emphasizes the significance of transovarial transmission in the dissemination of *B. ovis*, as the parasite demonstrates notable adaptation to the vector and persists within the tick for numerous successive generations (Markov and Abramov, 1970). Further research is warranted to explore additional factors influencing the transmission dynamics of *B. ovis* and to develop targeted control ,strategies to mitigate the impact of this disease on sheep populations.

## Data availability statement

The original contributions presented in the study are included in the article/supplementary material. Further inquiries can be directed to the corresponding author.

## Ethics statement

This study was carried out according to the regulations of animal and welfare issued by the Turkish legislation for the protection of animals. All animal experiments were approved by the Firat University, Animal Experiment Ethic Committee, protocol number 2023/12-05. The studies were conducted in accordance with the local legislation and institutional requirements. Written informed consent was obtained from the owners for the participation of their animals in this study.

## Author contributions

RF: Conceptualization, Investigation, Methodology, Project administration, Writing – original draft, Writing – review & editing. MU: Conceptualization, Investigation, Methodology, Project administration, Writing – original draft, Writing – review & editing. MA: Conceptualization, Investigation, Methodology, Project administration, Writing – original draft, Writing – review & editing. OC: Methodology, Project administration, Writing – original draft, Writing – review & editing. FS: Methodology, Writing – original draft, Writing – review & editing. RB: Formal analysis, Writing – original draft, Writing – review & editing. CS: Formal analysis, Writing – original draft, Writing – review & editing. SO: Conceptualization, Investigation, Methodology, Project administration, Writing – original draft, Writing – review & editing.
